# Use of a Minimal Microbial Consortium to Determine the Origin of Kombucha Flavor

**DOI:** 10.3389/fmicb.2022.836617

**Published:** 2022-03-21

**Authors:** Thierry Tran, Kevin Billet, Berta Torres-Cobos, Stefania Vichi, François Verdier, Antoine Martin, Hervé Alexandre, Cosette Grandvalet, Raphaëlle Tourdot-Maréchal

**Affiliations:** ^1^UMR Procédés Alimentaires et Microbiologiques, Institut Agro Dijon, Equipe Vin Alimentation Micro-Organismes Stress (VAlMiS), Université de Bourgogne Franche-Comté, Dijon, France; ^2^Departament de Nutrició, Ciències de l’Alimentació i Gastronomia, Institut de Recerca en Nutrició i Seguretat Alimentària (INSA), Universitat de Barcelona, Barcelona, Spain; ^3^Biomère, Paris, France

**Keywords:** kombucha, interaction, metabolites, sensory, volatile compounds, flavor, yeasts, acetic acid bacteria

## Abstract

Microbiological, chemical, and sensory analyses were coupled to understand the origins of kombucha organoleptic compounds and their implication in the flavor of the kombucha beverage. By isolating microorganisms from an original kombucha and comparing it to monocultures and cocultures of two yeasts (*Brettanomyces bruxellensis* and *Hanseniaspora valbyensis*) and an acetic acid bacterium (*Acetobacter indonesiensis*), interaction effects were investigated during the two phases of production. 32 volatile compounds identified and quantified by Headspace-Solid Phase-MicroExtraction-Gas Chromatography/Mass Spectrometry (HS-SPME-GC/MS) were classified according to their origin from tea or microorganisms. Many esters were associated to *H. valbyensis*, while alcohols were associated to both yeasts, acetic acid to *A. indonesiensis*, and saturated fatty acids to all microorganisms. Concentration of metabolites were dependent on microbial activity, yeast composition, and phase of production. Sensory analysis showed that tea type influenced the olfactive perception, although microbial composition remained the strongest factor. Association of *B. bruxellensis* and *A. indonesiensis* induced characteristic apple juice aroma.

## Introduction

When tasting a beverage, olfaction precedes the gustation itself, which establishes the odor as a key organoleptic component during the consumption of a product. Kombucha, a sour non-alcoholic fermented beverage obtained from sugared tea infusion is no exception. However, kombucha producers face difficulties with the control of kombucha’s stability of organoleptic quality including the olfactive profile, amid a very dynamic global market development (Kim and Adhikari, 2020). This is due to several factors among which: the absence of standard production procedure, highly biodiverse composition in yeasts and bacteria consortia in kombucha cultures, the lack of knowledge regarding the product itself, and the process parameters impacting its composition and organoleptic quality (Tran et al., 2020b; Harrison and Curtin, 2021). This also applies to the organoleptic stability of those products when they are put on the market without microbiological stabilization.

Indeed, the main established knowledge regarding kombucha is the symbiotic biological acidification process that constitutes the first phase of the production process in an open vessel. Yeasts provide available carbon substrate to acetic acid bacteria by hydrolyzing sucrose into monosaccharides and producing ethanol through alcoholic fermentation. In turn, acetic acid bacteria convert glucose into gluconic acid and ethanol into acetic acid through oxidative metabolism (Tran et al., 2020a). In parallel, acetic acid bacteria generate a cellulosic biofilm (pellicle) at the air/broth interface. Once a satisfactory degree of acidity is achieved it is possible to perform natural carbonation as a second production phase by bottling the product. As a consequence, this stimulates yeast alcoholic fermentation and allows accumulation of carbon dioxide and unfortunately also ethanol (Talebi et al., 2017). However, little is known about the contribution of the metabolism of kombucha microorganisms in volatile compounds production, besides the evident vinegary note brought by acetic acid (Tran et al., 2020b).

Some studies helped to understand the management of the main non-volatile parameters related to taste (meaning residual sugar and acidity) (Chen and Liu, 2000; Chakravorty et al., 2016; Tran et al., 2020a), but few studies have attempted to analyze kombucha’s volatile composition (Savary et al., 2021; Zhang et al., 2021) by providing first insights into the type and quantity of volatile compounds (aldehyde, ketones, alcohols, fatty acids and esters mainly). In the study of Zhang et al. (2021), Gas Chromatography/Mass Spectrometry (GC/MS) was performed on 6-day kombuchas and led to the detection of 22 volatile compounds (including ethyl octanoate, ethyl guaiacol and phenylethyl ethanol) common to all samples. Variations in composition between kombuchas made from different tea types appeared to involve only tea compounds and no metabolites produced by microorganisms. In the study of Savary et al. (2021), evolution of volatile composition of black tea kombucha was followed during 27 days of production. Clustering analysis showed an evolution of volatile profile with an increase in diversity from day 7 and peaking between day 11 and day 14. Produced compounds were fatty acids (acetic acid, short and medium chain fatty acids), alcohols (butanol, methylbutanol, methylpropanol, hexanol), and esters (ethanol, ethyl acetate, ethyl esters of short and medium chain fatty acids). Aldehydes (methylbutanal, heptanal, nonanal) were represented during the first days and ketones (heptanone, hexanone) during the last days of the process. Despite the several studies that performed descriptive sensory analysis (Neffe-Skocińska et al., 2017; Ivanišová et al., 2019; Zhang et al., 2021), no traditional descriptive sensory analysis using trained panel has been used. Untrained panel size varied between 16 and 60 individuals. Besides hedonic evaluations, descriptive analyses involved descriptors related to the sight (“color intensity”, “clarity”, “turbidity”, “darkness”), to the smell (“tea”, “lemon”, “acetic acid”, “yeast”, “fruity”, “floral”, “herbal”, “medicinal”) and to the taste (“acid/acidity”, “sweetness”, “bitter”). The sense of touch was once investigated with the descriptor “stinging”. Sourness or acidity appeared to be a fundamental descriptor of kombucha regarding the taste, whereas, the acetic acid, fruity, herbal and tea notes were characteristic of the smell. Coupled GC-MS and sensory analysis reported adequation of sweetness with residual sugar content and hypothesized on the link between isovaleric acid concentration and “unpleasant acidity”.

The present study aimed to investigate the topic of kombucha flavor by coupling microbiology, analytical chemistry, and sensory analyses. The objectives were to verify several hypotheses regarding the origins of kombucha flavor and how it is impacted by process parameters. Is kombucha’s olfactive profile affected by its microbial composition? If yes, is kombucha’s sensory components the result of microbial interactions? Do the secondary natural carbonation production phase or tea type play a role on sensory components? To achieve that, a similar approach to our previous study was conducted (Tran et al., 2020a). It consisted of comparing an original kombucha consortium with monocultures and cocultures of yeasts and acetic acid bacteria previously isolated from the original consortium. Thus, yeasts – acetic acid bacteria interactions as well as yeast – yeast interactions were investigated and included a condition gathering two yeasts and one acetic acid bacteria. Additionally, both production phases were investigated and the matrix effect was evaluated by using both black and green teas in association to the original kombucha culture used in the study.

## Materials and Methods

### Generation of Kombucha, Monocultures and Cocultures of Yeasts, and Acetic Acid Bacteria Isolated From Kombucha

Sugared teas and traditional kombuchas were produced according to our previous work (Tran et al., 2020a) with some modifications. First, 1% (m/v) of tea was steeped for 1 h, then 50 g of sucrose was added. Black tea (Pu’er Grade 1 TN4107) and green tea (Sencha Zhejiang TV4217) were used for kombucha production to investigate the matrix effect. Both teas were sourced at *Les Jardins de Gaïa* (Wittisheim, France). Sugared black tea (SBT) and sugared green tea (SGT) were obtained and used to produce black tea kombucha (BTK) and green tea kombucha (GTK) from the same kombucha culture. After cooldown to room temperature, 12% (v/v) of 7 days black tea kombucha broth was added. A primary inoculum was produced using the same procedure by using a mother culture obtained from the company Biomère (Paris, France) to ensure a physiological state of microorganism comparable to regular industrial scale production. The mother culture is a kombucha culture that was refreshed monthly with sugared black tea.

Yeasts and acetic acid bacteria strains were isolated from the broth of the black tea kombucha previously mentioned. Those microorganisms were selected for the present study according to their representation in terms of population during the elaboration of kombucha and their functionality (Tran et al., 2020a). The selection included *B. bruxellensis*, *H. valbyensis* and *A. indonesiensis*. The different modalities of monocultures and cocultures are detailed in Table 1.

**TABLE 1:** Description of the different cultures.

	Monocultures in sugared black tea	Yeast-Yeast coculture in sugared black tea	Yeast(s)-acetic acid bacteria cocultures (minimal consortia) in sugared black tea	Black or green tea kombucha (detected microorganisms)
Microbial composition (Code)	*B. bruxellensis* (BB) *H. valbyensis* (HV) *A. indonesiensis* (AI)	*B. bruxellensis* and *H. valbyensis* (BBHV)	*B. bruxellensis* and *A. indonesiensis* (BBAI) *H. valbyensis* and *A. indonesiensis* (HVAI) *B. bruxellensis*, *H. valbyensis* and *A. indonesiensis* (T)	*B. bruxellensis, H. valbyensis, Saccharomyces cerevisiae*, and bacteria including *A. indonesiensis* (BTK, GTK)

The association of at least one yeast and one acetic acid bacteria (BBAI, HVAI and T) can be seen as simplified kombucha consortium and will be referred to “minimal consortium”.

To generate precultures, yeasts and the acetic acid bacterium were restreaked on Wallerstein Lab (WL) agar (Hall, 1971) and De Man Rogosa and Sharpe (MRS) agar, respectively, from stocks kept at −20°C and incubated at 28°C. Yeasts and acetic acid bacteria isolates were inoculated in YPD liquid medium and MRS medium for yeasts and the bacterium, respectively. Those precultures were incubated at 28°C in static conditions for 2 days in sterile vessels with untight caps to allow gas exchanges. After incubation, cells were washed with sugared black tea then centrifugated (3,000 *g*, 10 min at 4°C). Each population was inoculated at the rate of 1.10^5^ cell mL^–1^ in sugared black tea only. This inoculation level was chosen because it matched those of the traditional process, as determined in our previous study (Tran et al., 2020a). Inoculation loading was determined through flow cytometry using a BD Accuri C6 (Franklin Lakes, NJ, United States) with 0.1 μg mL^–1^ propidium iodide in ultrapure water from Thermo Fischer Scientific (Waltham, MA, United States) to evaluate non-viable cell proportion (Stiefel et al., 2015). Cultivation of microorganisms using preculture in nitrogen rich growth media was a necessary step to reach desired inoculation level. The discrepancy in available nitrogen substrate in sugared tea may influence the microbial behavior in comparison to original kombuchas.

Cultures occurred in triplicates in 123 mL Boston flasks with a Specific Interfacial Surface (SIS; Cvetković et al., 2008) of 0.01 cm^–1^ with bottlenecks loosely covered with tin foil to allow gas exchanges during first 7 day phase (P1) of production. Then, flasks were fully closed for five more days during the natural carbonation phase (P2).

### Microbiological Analysis

Plate counting was performed to determine the populations of yeasts and/or acetic acid bacteria after inoculation (d0), at the end of P1 after 7 days (d7) and at the end of P2 after 12 days (d12). Despite the apparent production of cellulose, due to the absence of consistent cellulosic pellicle in cultures other than kombucha, microbiology data regarding the kombucha pellicles were not included. Successive decimal dilutions of samples on WL agar for yeasts and MRS agar for bacteria, respectively, were performed in triplicate. Characterization of the yeasts on WL agar plates allowed the quantification of yeasts subpopulation according to the species. This characterization was based on the linking of the morphological aspects with identities determined by 26S PCR on a representative number of isolates obtained from the kombucha culture, as described previously (Tran et al., 2020a).

### Analysis of Non-volatile Compounds

Endpoint chemical analyses at d0, d7 and d12 were performed on supernatants obtained by centrifugation (3,000 *g*; 15 min, 4°C) and kept frozen at -20°C. Total acidity was determined by titration with 0.1 N NaOH and 0.2% phenolphthalein as color indicator. pH values were measured with a Mettler Toledo Five Easy pH meter coupled with a LE498 probe. Acetic, lactic, malic, succinic acids concentrations were determined by HPLC as previously described (Tran et al., 2020a). Sucrose, glucose and fructose concentrations were determined using enzymatic kits from Biosentec (Auzeville-Tolosane, France).

### Analysis of Volatile Compounds

Volatile compounds were analyzed using Headspace-Solid Phase-MicroExtraction-Gas Chromatography/Mass Spectrometry (HS-SPME-GC/MS). Volatile compounds were extracted by Solid Phase Microextraction using a Combi-pal autosampler (CTC Analytics, Zwingen, Switzerland). An aliquot of 2 mL of sample spiked with 4-methyl-2-pentanol (Internal standard, 400 μg L^−1^) was placed into a 10 mL vial kept at 40°C under constant agitation. After 10 min of sample conditioning, a divinylbenzene/carboxen/polydimethylsiloxane (DVB/CAR/PDMS) SPME fiber (2 cm length, 50/30 μm film thickness) provided by Supelco (Bellefonte, PA, United States) was exposed to the sample headspace for 30 min. Then, it was desorbed in the gas chromatograph (GC) injection port at 260°C for 10 min.

Volatile compounds were analyzed by gas chromatography coupled to quadrupolar mass selective spectrometry using an Agilent 6890N Network GC system coupled to a quadrupolar mass selective analyzer Agilent 5975C Inert MSD (Agilent Technologies, Santa Clara, CA, United States) using helium as carrier gas, at a flow of 1.5 mL min^−1^. Analytes were separated on a Supelcowax-10 capillary column (60 m × 0.25 mm i.d., 0.25 μm film thickness) (Supelco, Bellefonte, PA, United States). The GC oven temperature was held at 40°C for the first 10 min, then increased to 150°C at 3°C min^−1^ and finally to 200°C at 15°C min^−1^, holding 5 min at that ending temperature. The temperature of the ion source and the transfer line were 200 and 275°C, respectively. Electron impact mass spectra were recorded at 70 eV ionization energy in full scan mode (m/z range from 35 to 300), 5.1 scans s^−1^.

Identification of compounds was carried out by comparison of their mass spectra and linear retention indices with those of standard compounds or with those available in mass spectrum library Wiley 6 and in the literature, respectively. Response factors of volatile compounds were calculated using a calibration curve obtained by analyzing different concentrations of reference compounds in the ranges 0.2–10000 μg L^−1^ (3-methylbutanal, ethyl acetate, butane-2,3-dione, 2-methylpropan-1-ol, 3-methylbutan-1-ol, 6-methyl-5-hepten-2-one, hexan-1-ol, 1-octen-3-ol, phenyl ethanol), 0.25–2.5 mg L^−1^ (3-methylbutanoic, non-anoic and decanoic acids) and 12.5–2500 mg L^−1^ (acetic acid). The quantitative assessment of organic acids was carried out in Extracted Ion Chromatogram (EIC) by selecting the m/z 60. The rest of compounds were quantified in the Total Ion Chromatogram (TIC). Reagents were purchased by Sigma-Aldrich (St Louis, MO, United States). Analytical repeatability, expressed as relative standard deviation (%), was assessed by analyzing 5 replicates of a quality control obtained by mixing different kombucha samples (Supplementary Material 1).

### Sensory Analysis

Descriptive sensory profiles were performed using a trained panel. Training occurred in two sessions. The first session included descriptor generation, qualitative and quantitative tasting, and smelling of standards. The second session included an evaluation of qualitative and quantitative perception of the standards followed by validation, and selection of 12 trained panelists. Selected descriptors and corresponding training standards with concentrations are described in Supplementary Material 2.

Sensory samples were produced using the same procedure as in part 2.1. but were produced later separately, kept unfiltered, decarbonated, and frozen (–20°C). The absence of filtration reflects the fact that kombucha is often sold unfiltered and the decarbonation eliminates the effect of carbon dioxide on the perception of the product both at visual and olfactive levels (Tran et al., 2020b). Thirty mL of thawed sample at room temperature was presented simultaneously to panelist in 50 cL white plastic beakers labeled with a 3-digits code. A latin square was used to eliminate bias of the order of tasting. A first evaluation session included the kombucha samples: BTKd7, GTKd7, BTKd12, GTKd12. A second evaluation session included the cultures in sugared tea: BBd7, HVd7, AId7, BBHVd7, BBAId7, HVAId7, Td7, BBAId12, HVAId12, and Td12. Each descriptor was evaluated using a horizontal intensity scale with 11 levels, going from 0 to 10, with 0 corresponding to “absence” and 10 to “very intense”. Samples were firstly evaluated on the olfactive descriptors, then on the gustative descriptors except the conditions BBd7, HVd7, AId7, and BBHVd7, which were only characterized on the olfactive level, since they did not correspond to minimal consortia and, therefore, they could not be comparable to kombuchas (original or issued from minimal consortium).

### Statistical Analyses

All biological samples were made in triplicate. Microbiological, non-volatile chemical composition, and sensory data sets were treated using univariate analysis. Average values were compared using ANOVA and Newman-Keuls pair test was applied in the case of significant difference (*p* < 0.05). In the case of sensory data, two-way ANOVA was performed. Statistics test, as well as multivariate analysis (MVA), were performed using R Statistical Computing software (v4.0.3), with the R studio interface (v 1.4.1106). Prior to MVA, variables were mean-centered and unit-variance was scaled. To visualize similarities and differences between samples, the FactoMineR package (v2.4) was employed to perform Principal Component Analysis. Hierarchical Cluster analyses (HCA) using the Ward minimum variance method was used to identify sub-groups among samples. The Fowlkes-Mallows (B_*K*_) similarity index was computed with the “dendextend” package (v1.14.0) and enabled the comparison between the sensory clustering and those obtained from GC-MS data set (Fowlkes and Mallows, 1983). Pair-wise Spearman correlations between metabolites abundancy and the sensory score were calculated with the “cor_test” function of statix package (v0.7.0). Correlation results and the significance of the interaction (*p* < 0.05) were showed as a heatmap using the pheatmap package (v1.0.12).

## Results and Discussion

### Microbiology and Non-volatile Composition

Population levels of yeasts and bacteria at day 7 (d7) and day 12 (d12) in the different cultures were determined using plate counting on selective and differential agar plates. The results are shown in Supplementary Material 3.

All cultures were conducted by inoculation of sugared tea at the rate of 1.10^5^ cells mL^–1^, except kombuchas which underwent traditional inoculation with 12% (v/v) kombucha from a previous batch. Initial populations for kombuchas (BTK and GTK) were 5.4.10^5^ and 9.0.10^4^ CFU mL^–1^ for total yeasts and total bacteria, respectively. The effect of microbial interactions has been assessed by comparing populations in cocultures compared to those in monocultures. At day 7 (end of P1), no significant differences among yeasts population could be observed among the cocultures except for *H. valbyensis* with significantly higher populations in cocultures when including *B. bruxellensis* (BBHVd7 and Td7) compared to kombuchas (BTKd7 and GTKd7; *p* < 0.05). However, the population of *A. indonesiensis* was significantly lower in all cultures compared to the monoculture (AId7), except when associated with *H. valbyensis* only (HVAId7; *p* < 0.05). Between day 7 and day 12 (end of phase P2) the population of *B. bruxellensis* increased significantly in kombuchas (BTK and GTK, respectively + 6.7.10^6^ and + 7.1.10^6^ CFU mL^–1^). The same increase was observed for *H. valbyensis* when in coculture with *B. bruxellensis* only (BBHV). Oppositely, a decrease of *A. indonesiensis* population occurred in the monoculture (AI) (-2.5.10^6^ mL^–1^) and in the coculture with *H. valbyensis* (HVAI) (-5.0.10^6^ CFU mL^–1^), thus erasing any significant difference with other culture including the bacterium. In kombuchas (BTK and GTK), the population of *S. cerevisiae* increased between 9.0.10^3^ and 1.0.10^4^ CFU ml^–1^, but it was not detected (inferior to 1.10^3^ CFU mL^–1^) at day 7. It is worth noting that the dominant yeast species was different between the kombucha issued from the minimal consortium T (for the “Trio” composed of two yeasts and one acetic acid bacteria) and the original kombuchas: *H. valbyensis* was the dominant species in the kombucha issued from the minimal consortium T whereas, *B. bruxellensis* was predominant in original kombuchas. It can be hypothesized that, the higher biodiversity in original kombuchas is the reason for this difference, for example through the presence of *S. cerevisiae*.

Microbial dynamics were influenced by the nature of the consortium, whether a minimal one or original kombucha culture, and impacted the identity of the dominant yeast species. The first phase of production P1 favored *A. indonesiensis* growth in monoculture or when associated with *H. valbyensis* only. The presence of *B. bruxellensis* appeared to cancel this effect, possibly due to substantial differences in metabolism between the two yeast species (Tran et al., 2020a). This effect disappeared at the end of the natural carbonation phase P2 that influenced positively the dominant yeast species in black and green tea kombuchas as well as the yeasts cocultures (BBHV). This could also be due to the inhibition of acetic acid bacteria induced by the limitation of oxygen access, if they were present.

Endpoint chemical analyses were performed at the end of each production phase, respectively, at day 7 and day 12. Initial sucrose, glucose, and fructose average concentrations in sugared teas (SBT and SGT) were 58.3 ± 0.9 g L^–1^, 0.3 ± 0.4 g L^–1^ and 0.4 ± 0.4 g L^–1^, respectively. The results are presented in Supplementary Material 4. No significant difference of sucrose concentrations at day 7 or day 12 were obtained with values between 39.1 and 56.1 g L^–1^ (Supplementary Material 4), except between BBAId7 and BBAId12 (respectively, 55.3 and 39.1 g L^–1^; *p* < 0.05). For all cultures between day 7 and day 12, sucrose was not fully hydrolyzed and the monosaccharides concentrations did not exceed 5 g L^–1^. This suggests a progressive consumption of monosaccharides obtained from sucrose hydrolysis. Moreover, the concentration of glucose was significantly higher for the *A. indonesiensis* monoculture (AI) at day 12 (4.9 g L^–1^) compared to other cultures where the bacterium was present (*p* < 0.05), which suggests a different kinetics of glucose consumption due to coculture with yeasts. Little effect of microbial composition, phase or tea type could be seen on the consumption of sugars, which was not the case in our previous studies (Tran et al., 2020a). However, process duration and initial sugar concentration were different (24 days instead of 12 days for the total process duration and 66.6 g L^–1^ of initial total sugars).

The acidity parameters (pH and total acidity) were measured (Supplementary Material 4) and showed significant differences between the cultures (*p* < 0.05). Initial pH average value of sugared teas was 6.90 ± 0.10 with an average total acidity below 1 meq L^–1^. All cultures underwent a decrease in pH value (*p* < 0.05). At day 7, the coculture of BBAI had a significantly lower pH than *B. bruxellensis* monoculture (BB) and was equivalent to the *A. indonesiensis* monoculture (AI) (respectively, 4.43, 4.75, and 4.36), although no difference in total acidity was noted. No other effect of interaction or matrix was observed at day 7. Oppositely, kombucha issued from the minimal consortium T possessed a significantly higher total acidity compared to BBHV (*p* < 0.05), which highlights the contribution of *A. indonesiensis* in the production of organic acids (+ 5.7 meq L^–1^), without significant effect on the pH value. Kombuchas (BTK and GTK) had significantly higher total acidity values (respectively, 20.7, 19.0 meq L^–1^) than minimal consortia at day 7 (ranging between 7.0 and 12.0 meq L^–1^). Between day 7 and day 12 an increase in total acidity occurred for all cultures except for the *H. valbyensis* monoculture (HV) (+ 9.3, + 8.7, + 10.3, + 8.7, + 7.7, + 8.0, + 20.0, + 9.3 meq L^–1^, for BB, AI, BBAI, HVAI, BBHV, T, BTK, and GTK, respectively). This increase was not systematically associated to a decrease of pH value (not the case for kombucha issued from minimal consortium T and GTK). This suggests that the production of organic acids continued even with reduced oxygen access during P2. This phenomenon has already been observed in our previous study (Tran et al., 2020a). At day 12, comparison of cultures of BB, HV, AI, and BBHV with BBAI, HVAI and kombucha issued from minimal consortium T, respectively, showed that yeasts-acetic acid bacterium association induced a significant increase of total acidity. It is consistent with the acetic acid bacteria’s expected role in a minimal consortium. However, there was no significant difference between cultures of BBAI, HVAI and T. Minimal consortia BBAI, HVAI, T and kombuchas (BTK and GTK) possessed total acidity average values at day 12 ranging between 15.7 and 40.7 meq L^–1^ with a more efficient acidification for original kombuchas. Differences in inoculation process must be considered here. Original kombuchas were traditionally inoculated with 12% (v/v) of a previous acidic broth. As a result, initial average total acidity of kombuchas were 5.0 ± 0.1 meq L^–1^ and appeared to favor the acidification process, whereas initial total acidity of minimal consortia was inferior to 1 meq L^–1^. Moreover, BTK pH value was significantly lower than GTK’s (3.69 and 4.00, respectively), in association with a significantly higher total acidity at day 12 (40.7 and 28.3 meq L^–1^, respectively), which indicates an effect of the matrix on the microbial activity without significant difference on the microbial populations (Supplementary Material 3).

Behavior in terms of sugar consumption and acidification were globally consistent with our previous study (Tran et al., 2020a). Minimal consortia, as well as kombuchas consumed sugars to produce organic acids more efficiently than monocultures, but the performance of minimal consortia was not as good as the kombuchas. Additionally, no meaningful difference in sugars consumption could be observed. Thus, the microbial composition appeared to play a significant role on the production process, with a more intense acidification of yeast(s)-acetic acid bacteria cultures, despite the decrease of *A. indonesiensis* population induced by the presence of *B. bruxellensis*. The transition from P1 to P2 impacted the microbial dynamics by increasing yeasts and decreasing *A. indonesiensis* populations due to limitation of oxygen access. In the original kombuchas, it led to increased populations of *S. cerevisiae*. Finally, the tea type did not influence the microbial dynamics but had an impact on the level of acidification at the end of P2. Those three factors: microbial composition, phase, and matrix clearly have an impact on the microorganisms’ dynamics and on the kombucha’s non-volatile composition. In contrast, the next part assesses the microbial role on the volatile composition.

### Volatile Composition

The analysis of the samples by HS-SPME-GC-/MS allowed the identification and quantification of 32 volatile metabolites belonging to different molecular families: alcohols, aldehydes, ketones, esters, phenol, and saturated fatty acids (including acetic acid; Table 2). Many metabolites such as phenylethyl ethanol, acetic acid, valeric and isovaleric acids were previously quantified in kombucha (Savary et al., 2021; Zhang et al., 2021). ANOVA was performed for the monocultures (BBd7, HVd7, AId7, BBd12, HVd12, and AId12) and SBT to determine if some metabolites were significantly more concentrated in one of those conditions (Supplementary Material 5). If a metabolite was detected in higher concentration in SBT, it was classified as “varietal”. If a metabolite was present in SBT and its concentration increased positively in one of the cultures, it was classified as “varietal and fermentative”. If a metabolite was not detected in SBT and produced in significant quantity in the monocultures, it was classified as “fermentative”. Signature metabolites were assigned to SBT of one microorganism if metabolites levels were significantly higher in one specific condition.

**TABLE 2:** Detected and quantified volatile metabolites with chemical, sensory and origin features (Luebke, 1980; Lambrechts and Pretorius, 2000).

Code	IUPAC name	Common name	Chemical Family	Origin	Microorganism and phase where metabolite acts as signature	Smell
m01	Ethanal	Acetaldehyde	Aldehyde	Fermentative	No association to either microorganisms or phases	Sour, green apple (Lambrechts and Pretorius, 2000)
m02	Methyl acetate	Methyl acetate	Ester	Varietal and fermentative	No association to either microorganisms or phases	Solvant (Luebke, 1980)
m03	Ethyl acetate	Ethyl acetate	Ester	Varietal and fermentative	HVd7, HVd12	Varnish, nail polish, fruity (Lambrechts and Pretorius, 2000)
m04	2-methylbutanal	2-methylbutanal	Aldehyde	Varietal and fermentative	No association to either microorganisms or phases	Malt (Luebke, 1980)
m05	3-methylbutanal	Isovaleraldehyde	Aldehyde	Varietal	No association to either microorganisms or phases	Warm, herbaceous, slightly fruity (Lambrechts and Pretorius, 2000)
m06	Ethanol	Ethanol	Alcohol	Fermentative	BBd7, BBd12, HVd7, HVd12	Alcohol (Lambrechts and Pretorius, 2000)
m07	Ethyl propanoate	Ethyl propanoate	Ester	Fermentative	HVd7, HVd12	Sweet fruity rum juicy fruit grape pineapple (Luebke, 1980)
m08	Ethyl 2-methylpropanoate	Ethyl isobutyrate	Ester	Fermentative	BBd12	Sweet, rubber (Luebke, 1980)
m09	Propyl acetate	Propyl acetate	Ester	Fermentative	HVd7, HVd12	Solvent-like pungency, lifting, fusel, amyl alcohol, sweet and fruity (Luebke, 1980)
m10	Pentan-2-one	2-pentanone	Ketone	Varietal	No association to either microorganisms or phases	Etherial, diffusive and sweet banana-like with fermented woody nuance (Luebke, 1980)
m11	Butane-2,3-dione	Diacetyl	Ketone	Fermentative	No association to either microorganisms or phases	Buttery (Lambrechts and Pretorius, 2000)
m12	Hexanal	Hexanal	Aldehyde	Varietal	No association to either microorganisms or phases	Grass, tallow, fat (Luebke, 1980)
m13	2-methylpropan-1-ol	Isobutanol	Primary alcohol	Fermentative	No association to either microorganisms or phases	Wine, solvent, bitter (Luebke, 1980)
m14	3-methylbutan-1-ol	Isoamylacetate	Ester	Fermentative	No association to either microorganisms or phases	Banana, pear (Lambrechts and Pretorius, 2000)
m15	2-methyl-2-propanol	Tert-butanol	Primary alcohol	Varietal and fermentative	No association to either microorganisms or phases	Camphor (Luebke, 1980)
m16	3-methylbutan-1-ol	Isoamyl alcohol	Primary alcohol	Varietal and fermentative	HVd7, HVd12	Marzipan (Lambrechts and Pretorius, 2000)
m17	3-hydroxybutan-2-one	Acetoin	Ketone	Fermentative	No association to either microorganisms or phases	Butter, cream (Luebke, 1980)
m18	6-methyl-5-hepten-2-one	6-methyl-5-hepten-2-one	Ketone	Varietal	No association to either microorganisms or phases	Fruity, apple, musty, ketonic and creamy with slight cheesy and banana nuances (Luebke, 1980)
m19	Hexan-1-ol	Hexanol	Primary alcohol	Varietal and fermentative	HVd7, HVd12	Resin, flower, green (Luebke, 1980)
m20	1-octen-3-ol	1-octen-3-ol	Secondary alcohol	Varietal and fermentative	BBd7, HVd7	Earthy, green, oily, vegetative and fungal (Luebke, 1980)
m21	Heptanol	Heptanol	Alcohol	Varietal and fermentative	HVd7, HVd12	Mushroom, green (Luebke, 1980)
m22	Benzaldehyde	Benzaldehyde	Aldehyde	Varietal and fermentative	No association to either microorganisms or phases	Bitter almond (Lambrechts and Pretorius, 2000)
m23	Nonan-1-ol	Nonanol	Alcohol	Varietal and fermentative	HVd7, HVd12	Fresh clean fatty floral rose orange dusty wet oily (Luebke, 1980)
m24	2-phenylethyl acetate	2-phenylethyl acetate	Ester	Varietal and fermentative	HVd7	Rose, honey, fruity, flowery (Lambrechts and Pretorius, 2000)
m25	2-phenylethan-1-ol	Phenylethanol	Alcohol	Varietal and fermentative	HVd12	Floral, rose (Lambrechts and Pretorius, 2000)
m26	Phenol	Phenol	Phenol	Varietal and fermentative	No association to either microorganisms or phases	Phenol (Luebke, 1980)
m27	Acetic acid	Acetic acid	Saturated fatty acid	Fermentative	AId12	Vinegar, pungent (Lambrechts and Pretorius, 2000)
m28	3-methylbutanoic acid	Isovaleric acid	Saturated fatty acid	Fermentative	No association to either microorganisms or phases	Rancid, cheese, sweaty, rancid, fatty, pungent (Lambrechts and Pretorius, 2000)
m29	Pentanoic acid	Valeric acid	Saturated fatty acid	Fermentative	HVd12	Unpleasant (Lambrechts and Pretorius, 2000)
m30	Octanoic acid	Caprylic acid	Saturated fatty acid	Fermentative	No association to either microorganisms or phases	Oily, fatty rancid, soapy, sweet, faint fruity, butter (Lambrechts and Pretorius, 2000)
m31	Nonanoic acid	Nonanoic acid	Saturated fatty acid	Fermentative	No association to either microorganisms or phases	Waxy, dirty and cheesy with cultured dairy nuance (Luebke, 1980)
m32	Decanoic acid	Capric acid	Saturated fatty acid	Fermentative	No association to either microorganisms or phases	Fatty, unpleasant, rancid, citrus, phenolic (Lambrechts and Pretorius, 2000)

Each class gathered different chemical families (Table 2). However, we can underline that saturated fatty acids were all classified as fermentative and that the yeasts’ signature metabolites were mainly marked by alcohols and esters, as it can be expected based on other matrices such as wine and cider (Mo et al., 2003; Hirst and Richter, 2016). Ethanol and 1-octen-3-ol appeared to be signature metabolites of both *B. bruxellensis* and *H. valbyensis*. Most metabolites were associated with *H. valbyensis* only (ethyl acetate, isoamyl alcohol, hexanol, heptanol, nonanol, 2-phenyl acetate, phenyl ethanol, ethyl propanoate, ethyl isobutyrate, propyl acetate). It has been reported in cider that *H. valbyensis* in coculture with *S. cerevisiae* enhanced the production of esters (ethyl acetate, phenylethyl acetate). Those compounds in pure solution are associated with solvent, floral, and fruity aroma (Lambrechts and Pretorius, 2000). Identified saturated fatty acids were small and medium-chain fatty acids produced during alcoholic fermentation and were implied in the biosynthesis of long chain fatty acids (Viegas et al., 1989; Stevens and Hofmeyr, 1993; Lambrechts and Pretorius, 2000; van Roermund et al., 2003). Medium-chain fatty acids were reported to have an inhibitory effect on the growth and fermentative activity of *S. cerevisiae* in conjunction with ethanol concentration and low pH value. Inhibition of lactic acid bacteria *Oenococcus oeni* by caprylic and lauric acids have also been reported in the context of interaction with *S. cerevisiae* during wine fermentation (Guilloux-Benatier et al., 1998). Little data is available for acetic acid bacteria (Yamada et al., 1981). However, release of medium-chain fatty acids was reported for lactic acid bacteria (Nakae and Elliott, 1965). According to the study of Chen et al. (1980), those compounds could be released during the autolysis of yeasts and could be involved in the “yeasty” aroma of lees. Indeed, acetic acid was the signature metabolite of *A. indonesiensis* (Tran et al., 2020a). Metabolites classified as varietal such as isovaleraldehyde, hexanal, benzaldehyde, heptanol, nonanol, and phenyl ethanol are typically found in tea (Ho et al., 2015). Aldehydes and ketones were mainly classified as purely varietal.

**FIGURE 1:**
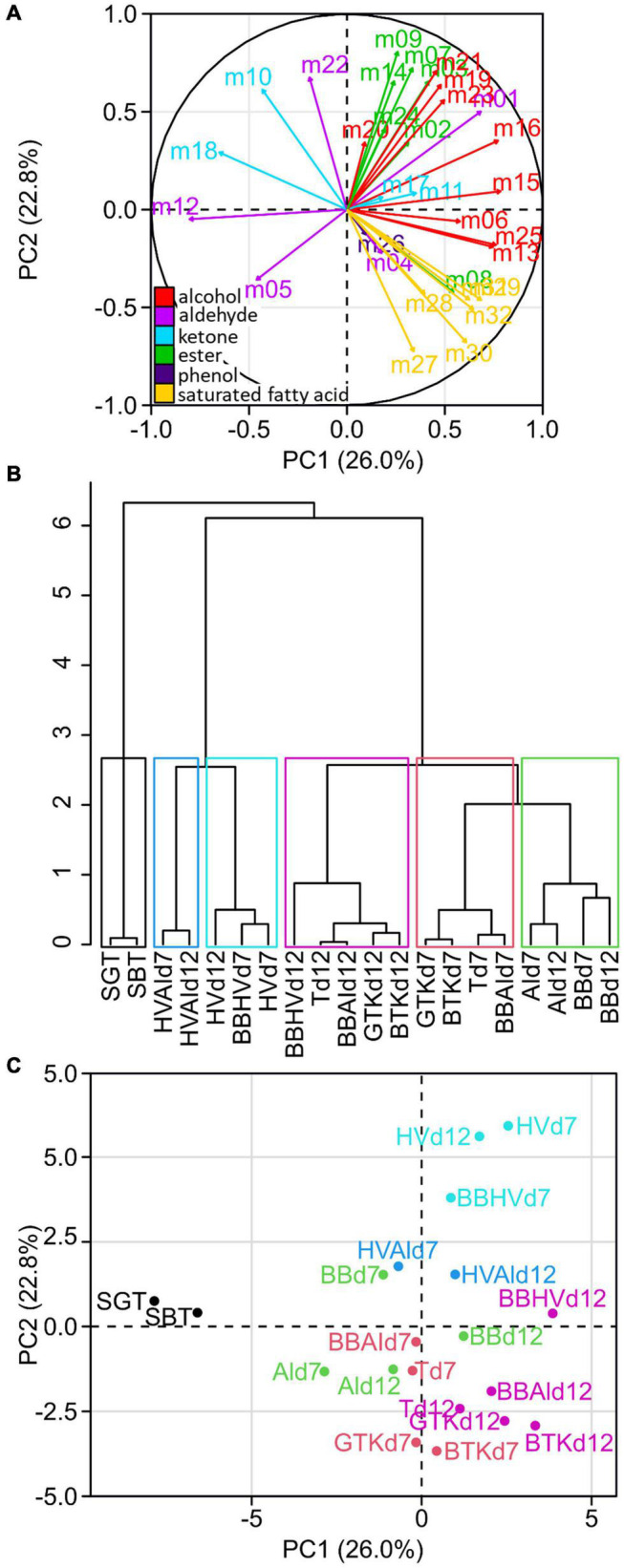
Unsupervised classification using principal component analysis (PCA) and hierarchical clustering (HCA) on the relative concentration of the 32 metabolites from the 18 cultures (*n* = 3). Loading plot **(A)** shows the projection of each metabolite numbered consistently with Table 2. The metabolite coloration corresponds to its chemical family: alcohol (red), aldehyde (magenta), ketone (blue), ester (green), phenol (purple) and saturated fatty acid (yellow). HCA **(B)** with cut-off enabling a separation in six clusters depicted in the PCA score plot **(C)** with each cluster associated to one color.

Each metabolite was labeled with a code (given in Table 2) and was used as parameter to visualize similarities between samples through PCA and HCA (Figure 1). The sum of eigenvalues of the dimension PCA1 (26.0%) and PCA2 (22.8%) was equal to 48.8%. The interpretation of those two dimensions was judged satisfactory to describe the phenomenon. The loading plot (Figure 1A) showed that metabolites vectors of the same family projected in similar PCA area, except aldehydes and ketones. Alcohols projected positively on the PC1 axis, esters positively on PC2, saturated fatty acid and phenol positively on PC1 and negatively on PC2. This result showed that the production of volatile metabolites occurred by chemical families for the three main ones, which were all fermentative metabolites (Table 2), involved possibly common metabolic pathways.

The HCA allowed the discrimination of samples into clusters of similar volatile compositions (Figure 1B). The first cluster gathered the sugared teas (SBT and SGT). The second cluster gathered coculture of *H. valbyensis* and *A. indonesiensis* (HVAId7 and HVAId12). The third cluster gathered *H. valbyensis* monocultures (HVd7 and HVd12) and BBHVd7. The fourth cluster gathered *B. bruxellensis* and *A. indonesiensis* monocultures (BBd7, AId7, BBd12, and AId12). The fifth cluster gathered minimal consortia including *B. bruxellensis* and the original kombuchas at day 7 (BBAId7, Td7, BTKd7, and GTKd7). The sixth cluster gathered the same cultures at day 12 (BBAId12, Td12, BTKd12, and GTKd12) and the coculture BBHVd12. The organization of the dendrogram’s branches enables to hierarchize the factors that affected the samples volatile composition. The main factor was the microbial activity (sugared teas against cultures) and separated the samples according to their volatile composition. Within the cultures, the effect of yeast composition separated secondarily the samples including *H. valbyensis* with and without the presence of *B. bruxellensis*, apart from BBHVd7 that seemed to be mainly influenced by the presence of *H. valbyensis*, in opposition to BBHVd12 that seemed influenced by *B. bruxellensis*. In the case of BBHV, the phase of production appeared to modify the microbial activities without effect on yeast species dominance (Supplementary Material 3). Therefore, associations can be made between *H. valbyensis* and P1, and between *B. bruxellensis* and P2. Indeed, the third effect of the production phase could be clearly observed for the minimal consortia including *B. bruxellensis* and the kombuchas, which were dominated by this yeast species (Supplementary Material 3). Finally, monocultures of *B. bruxellensis* and *A. indonesiensis* were closer to the minimal consortia including *B. bruxellensis* (BBAI and T), which highlighted a fourth effect of yeast-acetic acid bacteria interaction. It is noteworthy that the difference in tea types induced no matrix effect on the clustering, both before and after microbial activity. So, regardless of the tea type, kombuchas were in the same clusters. The cultures that showed the closest similarity to the kombuchas were the cocultures including at least both *B. bruxellensis* and *A. indonesiensis* both at day 7 and day 12, with or without the presence of *H. valbyensis* (BBAI and T). Consequently, the minimal presence of *B. bruxellensis* and *A. indonesiensis* defined a functional consortium on the level of volatile composition. Interestingly, this echoes the results of our previous study (Tran et al., 2020a), where this couple of microorganism was also a suitable combination for a functional consortium regarding acidification. The major volatile metabolites found in those clusters besides ethanol and acetic acid were ethyl acetate and isovaleric acid with concentrations ranging from 600 to 7000 μg L^–1^.

Figure 1C presents the PCA’s score plot and demonstrates the association of clusters to chemical families in relationship to the PCA loading plot (Figure 1A). Sugared teas, located on the left part of the plot, were associated with ketones and aldehydes that were mainly signature metabolites of SBT. Clusters with minimal consortia including *B. bruxellensis* and kombuchas were clearly associated with the production of alcohols and saturated fatty acids, while cultures involving *H. valbyensis* without *B. bruxellensis* (HV, HVAI) and BBHVd7 were associated to the production of alcohols and esters.

More details can be obtained from the ANOVA of minimal consortia (BBAI, HVAI and T) and BTK (Supplementary Material 6). *H. valbyensis* significantly contributed on signature metabolites in Td7 (ethyl acetate, ethyl isobutyrate and isoamyl alcohol) compared to BBAId7 (*p* < 0.001), but this contribution sustained only with ethyl isobutyrate and isoamyl alcohol at day 12. Therefore, *H. valbyensis* could possess a functionality in the minimal consortium T by enrichment of ester and alcohols of the volatile profile and potentially the olfactive profile of the product. Moreover, for all cocultures, the minimal consortia and SBT, P2 significantly increased the concentrations of isobutanol and isoamyl alcohol (*p* < 0.001), which became major volatile compounds (between 700 and 2000 μg L^–1^). This could be the consequence of redox potential regulation due to restricted oxygen access (Dijken and Scheffers, 1986; Quain, 1988; Hazelwood et al., 2008).

By comparing monocultures and cocultures at day 7 using ANOVA (Supplementary Material 7), it is possible to observe that the presence of *B. bruxellensis* and/or *A. indonesiensis* decreased the production of ethyl propanoate, isoamyl alcohol, hexanol, heptanol, and nonanol by *H. valbyensis*. Two hypotheses can be made: either the microorganisms competed for the substrate or precursor needed to produce those metabolites, or the metabolites were used by the other microorganisms (commensalism) (Ivey et al., 2013). Namely, the presence of *A. indonesiensis* significantly decreased the concentration of alcohols (ethanol, isobutanol, hexanol, nonanol, *p* < 0.01). For example, nonanol could be oxidized into nonanoic acid in the same manner that ethanol is oxidized in acetic acid or glycerol in dihydroxyacetone (Matsushita et al., 1994), although no specific enzyme for the conversion of nonanol has been yet reported. Consequently, the functionality of *H. valbyensis* was limited by the presence of other microorganisms due to microbial interactions. Additionally, it is worth noting that pure varietal metabolites either showed no significant variation linked to microbial activity (2-pentanone, 6-methyl-5-hepten-2-one) or showed significant decrease regardless of microbial composition (isovaleraldehyde, hexanal; *p* < 0.001).

**FIGURE 2:**
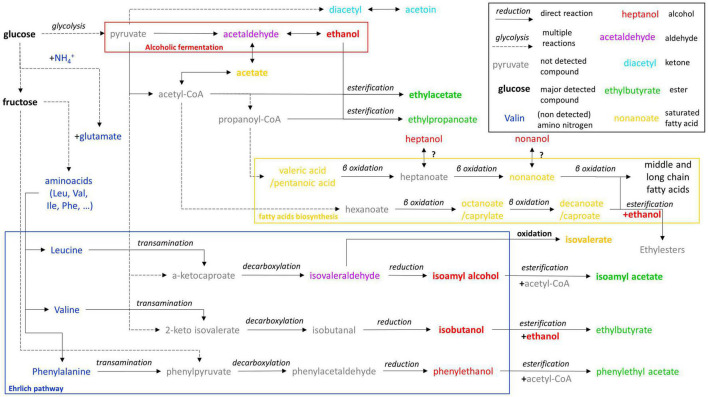
Putative metabolic pathways of fermentative volatile metabolites detected based on *S. cerevisiae* metabolism (Yoshizawa et al., 1961; Ardö, 2006; Ljungdahl and Daignan-Fornier, 2012; Yu et al., 2016; Belda et al., 2017).

A putative metabolic scheme based on metabolic pathways of *S. cerevisiae* was described in the literature and KEGG database (Kanehisa, 2000) and gathered most of fermentative metabolites identified in the present study (Figure 2). It highlights the metabolic pathways that are dependent on sugar (glucose and fructose) and those dependent on nitrogenous substrates (ammonium NH_4_^+^ and amino acids). Production of volatile compounds in wine for example are mainly attributed to the Ehrlich pathways that rely on the transamination of an amino acid, the decarboxylation of the keto acid (phenylpyruvate in the case of phenylalanine metabolism), then the reduction of the acid into volatile alcohol (Ehrlich, 1907). The alcohols can then be esterified to produce volatile esters (Belda et al., 2017). However, keto acids and phenylpyruvate can be produced from pyruvate using amino acids biosynthesis pathways (Kanehisa, 2000; Yu et al., 2016). Consequently, volatile alcohols such as isoamyl alcohol, isobutanol, and phenylethanol and their corresponding esters, can be produced independently from the corresponding amino acids (leucine, valine, phenylalanine). Other metabolites unrelated from the Ehrlich pathway can be produced solely from glucose (ethyl esters, ketones, saturated fatty acids). Although amino acids can be synthetized *de novo* from ammonium (Ljungdahl and Daignan-Fornier, 2012), the initial amount of free amino nitrogen in tea infusion (63 μg L^–1^ in our previous study: Tran et al., 2020b) is insufficient to produce the amount of volatile metabolites quantified in the present study. Moreover, no volatile metabolite absolutely dependent on the Ehrlich pathways were detected in the present study, nor in recent studies (Savary et al., 2021; Zhang et al., 2021). In these studies, the detection of ethyl esters of caprylic and caproic acids tends to corroborate this hypothesis. Therefore, the volatile profile of kombucha appears to be determined by the composition of the sugared tea infusion matrix, that consists in a low N/C ratio. For example, N/C ratio of sugared teas in the present study was around 1.3.10^–6^, which is 577 times lower than the N/C ratio of a grape must of 200 g L^–1^ sugars and 150 mg L^–1^ of assimilable nitrogen (7.5.10^–4^).

The volatile profile of kombucha appeared to be mainly impacted by the microbial composition and interactions. The production phase induced changes in the volatile profile. Tea type had little effect on it. Also, the volatile compounds of kombucha are suspected to be determined by the particular composition of the sugared tea matrix in terms of carbon and nitrogen substrates availability. However, the real implications of those effects on the product can only be evaluated through sensory analysis.

### Linking Volatile Composition to Sensory Profiles

Among the 20 conditions analyzed by GC/MS, 14 underwent descriptive sensory analysis through the evaluation of newly produced samples by 12 trained panelists. The samples gathered minimal consortia and kombuchas at day 7 and day 12, and monocultures at day 7.

ANOVA revealed no significant difference in sweetness, bitterness, and astringency between the samples (Supplementary Material 8). In contrast, sourness was significantly higher for BBAI and HVAI samples at day 12 than at day 7 (*p* < 0.001). Regarding kombuchas only, sweetness and sourness reached the highest scores (respectively, 5.5 and 3.7), whereas bitterness and astringency showed lower scores (1.9 and 2.1), which clearly established the sweetness and sourness balance as main gustative parameters of kombucha and probably mask bitterness and astringency of tea as previously hypothesized (Tran et al., 2020b).

**FIGURE 3:**
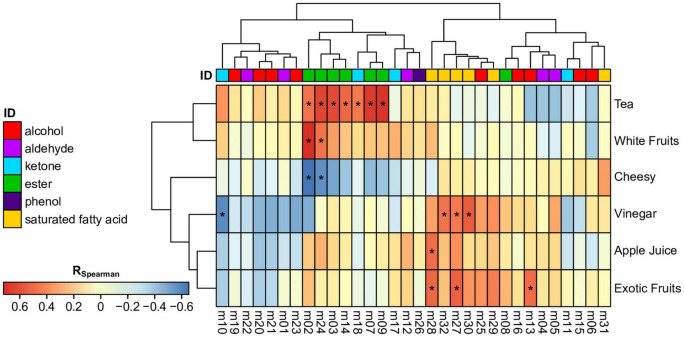
Heatmap depicting pair-wise Spearman correlations (R; *p* < 0.05: *) between a sensory descriptor score and a single metabolite numbered as in the Table 2 and colored consistently as its chemical family.

Volatile compositions were linked to olfactive sensory scores per descriptor using pair-wise Spearman correlations (Figure 3). The results showed significant correlations of esters and ketones with tea (ethyl acetate, isoamyl acetate, 6-methyl-5-hepten-2-one, ethyl propanoate, propyl acetate; *R* > 0.54, *p* < 0.048) and together with white fruits (methyl acetate, 2-pheynlethyl acetate; *R* > 0.55, *p* < 0.042). Consistently, those compounds are generally associated to fruity aroma (Lambrechts and Pretorius, 2000). All those metabolites are varietal and most of them contribute to the fermentative signature of *H. valbyensis*. This signifies that those metabolites enhanced the tea and white fruits aroma which could be a positive contribution to the olfactive profile. In contrast, saturated fatty acids and alcohols are involved in the expression of vinegar, apple juice, and exotic fruits aroma. Together with decanoic and octanoic acid, acetic acid expectedly contributed to the vinegar aroma (*R* > 0.54, *p* < 0.045) whereas the pure varietal 2-pentanone was negatively correlated (*R* = -0.58; *p* = 0.029). Acetic acid also played a significant role in exotic fruits aroma with isovaleric acid and isobutanol (*R* > 0.56; *p* < 0.040). Additionally, isovaleric acid is significantly correlated to the apple juice aroma (*R* = 0.56, *p* = 0.036). Besides acetic acid, the odor of those compounds in pure solutions are generally associated with oily, fatty and rancid notes, and isobutanol with solvent (Lambrechts and Pretorius, 2000). The correlation with fruity odors must then be the result of sensory interaction between the compounds involved on global perception (Chambers and Koppel, 2013). Finally, the cheesy aroma was significantly correlated only negatively with methyl acetate and 2-phenylethyl acetate (*R* < -0.56, *p* < 0.042). This might be related to a masking effect on fruity aroma.

**FIGURE 4:**
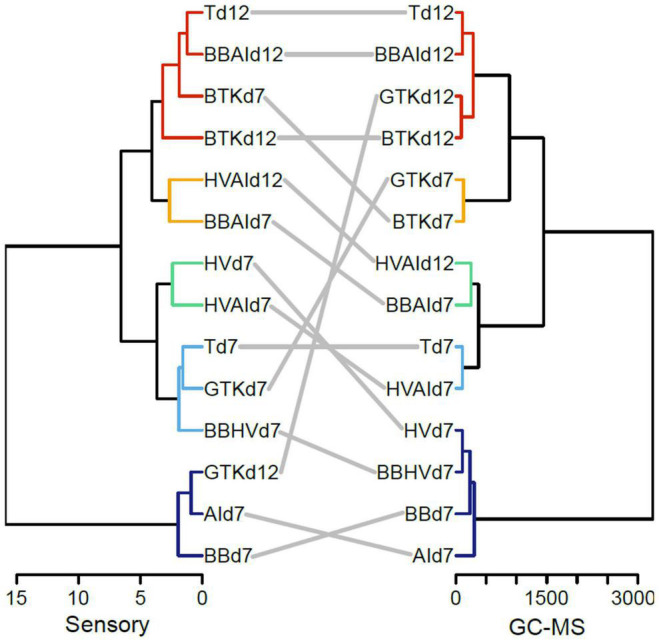
Dendrogram comparison based on Ward clustering performed on sensory scores **(left)** and volatile metabolite concentrations **(right)** in the 32 detected metabolites among the 14 cultures evaluated by sensory analysis.

Figure 4 displays a dendrogram comparison of the 14 conditions treated by GC-MS and by sensory analyses. To go further on the similarity between the GC-MS and sensory dendrograms, the Fowlkes-Mallows index (B_*K*_) was used. The B_*K*_ index is linked to the *k* cut-off threshold used for both dendrograms and to the number of matching entries in the resulting *k* clusters of each tree. The resulting B_*K*_ plot (Supplementary Material 9) shows increased similarity for *k* clusters between 4 and 5 and thus indicates relationships between volatile composition and sensory perception. Differences in clustering were visible but the general organization remained similar with separation of *B. bruxellensis* and *A. indonesiensis* monocultures from cocultures and kombucha. Also, HVAId12 and BBAId7 samples remained in the same clusters. The conservation of the clustering of Td12 with BBAId12 and BTKd12 samples was striking and showed the closeness of functional consortia and kombucha in volatile composition carried on to the olfactive profile. Considering the sensory dendrogram alone, monocultures were separated from cocultures except for *H. valbyensis* monoculture that was related to HVAId7 sample. Within the coculture and kombucha branch, cultures dominated by *H. valbyensis* (HVAId7, HV, Td7, and BBHVd7) shared closeness (Supplementary Material 3). A striking difference involved GTKd7 sample that was separated to BTKd7 sample. This suggests a matrix effect that was not expressed through GC-MS volatile compounds analysis. It is likely that some organoleptic varietal compounds specific to green tea were not detected. GTKd12 sample was related to *B. bruxellensis* and *A. indonesiensis* monocultures (BBd7 and AId7) due to unexpected intense cheesy off-flavor. Those three samples showed significantly higher cheesy notes (*p* < 0.001) (Supplementary Material 8). In contrast, *H. valbyensis* (HV) monoculture and HVAId7 sample showed significantly higher tea aroma compared to BBd7, AId7 and GTKd12 samples (*p* < 0.001), confirming the masking effect of the cheesy note. Interestingly, BBAId7 showed higher white fruits and apple juice notes than BBd7 and AId7 samples. This worked similarly with HVAId7, HVd7, and AId7 samples for the apple juice aroma only. Therefore, the characteristic apple juice aroma of kombucha appears to result from the yeast-acetic acid bacteria interaction (Tran et al., 2020b). Moreover, this interaction relied on sensory interaction of compounds produced by each microorganism and not from compounds issued from microbial interaction.

In contrast with the volatile composition, the production phase had less effect on the sensory profile of the samples, while the tea type appeared to induce a matrix effect. The effect of microbial composition remained dominant and the simultaneous presence of *B. bruxellensis* and *A. indonesiensis* was confirmed to be key for the typicity of kombucha’s olfactive profile that includes the apple juice note.

## Conclusion

Through this study coupling microbiology, analytical chemistry, and sensory analysis, many questions regarding the origins of kombucha’s organoleptic components could be answered. Olfactive profiles of cultures and kombuchas were clearly associated to volatile profiles and identified the association of at least one yeast with the tested acetic acid bacterium, such as *B. bruxellensis* and *A. indonesiensis* as minimal functional consortium to produce the closest beverage to kombucha. This statement was previously made for the main non-volatile compounds of kombucha (organic acids, sugars, and ethanol; Tran et al., 2020b). The typical apple juice aroma of kombucha emerged from yeast-acetic acid bacteria association as well. *H. valbyensis*’ contribution in the olfactive and volatile profiles was beneficial in terms of quality, with a potential enhancement of fruity aroma through the production of esters but it was limited because of interactions with other microorganisms and the secondary natural carbonation phase. This production phase also stimulated the production of alcohols.

This study contributed to better understand the kombucha’s volatile composition in terms of origins (varietal and/or fermentative). It also revealed a new hypothesis about kombucha’s composition determinism and its relationship with the sugared tea matrix composition. Ultimately, a matrix effect could be observed on the sensory level although it had the least impact on the composition in detected volatile compounds. Those results should enable further investigation to understand, improve, and control the organoleptic quality of kombucha. Namely, the influence of acetic acid bacteria or lactic acid bacteria species and their interactions would constitute research topics of high interest.

## Data Availability Statement

The raw data supporting the conclusions of this article will be made available by the authors, without undue reservation.

## Author Contributions

TT took the lead of the writing of this article, but all other authors provided critical and complementary elements to the manuscript, performed microbiological and non-volatile chemical analyses, as well as ANOVA of volatile compounds and sensory analyses. KB performed statistical treatment and figures conception of PCA, HCA, heatmap, dendrogram comparison analysis and Bk-plot. BT-C and SV performed HS-SPME-GC-MS analysis of volatile compounds. FV and AM provided the kombucha cultures used in the experiments. All authors contributed to the article and approved the submitted version.

## Conflict of Interest

FV and AM are the leaders of the company Biomère. The remaining authors declare that the research was conducted in the absence of any commercial or financial relationships that could be construed as a potential conflict of interest.

## Publisher’s Note

All claims expressed in this article are solely those of the authors and do not necessarily represent those of their affiliated organizations, or those of the publisher, the editors and the reviewers. Any product that may be evaluated in this article, or claim that may be made by its manufacturer, is not guaranteed or endorsed by the publisher.
