# Supplementary material for: Use of a Minimal Microbial Consortium to Determine the Origin of Kombucha Flavor
Source: Front Microbiol. 2022 Mar 21;13:836617. doi: 10.3389/fmicb.2022.836617 (PMC8978889; doi:10.3389/fmicb.2022.836617)

**Supplementary Material S2: Descriptors and associated standards with concentrations for sensory analysis and panel training.**

| **Descriptor** | **Standard** | **Concentrations** |
| --- | --- | --- |
| Sweetness | Sucrose | 10-50 (g L^-1^) |
| Sourness | Lactic acid | 0.045-0.36 (g L^-1^) |
| Bitterness | Caffein | 1.5 (g L^-1^) |
| Astringent | Grape tannins | 0.6 (g L^-1^) |
| Vinegar | Acetic acid | 0.3-2.4 (g L^-1^) |
| Tea | Black and green tea infusion from Les jardins de Gaïa (Wittisheim, France) at room temperature | 1% (m/v) |
| White fruit | As an example, quince standard #11 from “Le Nez du Vin”, Editions Jean Lenoir (Paris, France) |  |
| Exotic fruit | As an example, pineapple standard #4 from “Le Nez du Vin”, Editions Jean Lenoir (Paris, France) |  |
| Apple juice | Commercial apple juice from Casino (Saint Etienne, France) |  |
| Cheesy | Organic Parmesan cheese from Casino (Saint Etienne, France) |  |

**Supplementary Material S3: Population levels expressed in CFU mL^-1^ in the different cultures at 7 (d7) and 12 days (d12) after inoculation (average values, n = 3).**

| Samples | *B. bruxellensis* | *H. valbyensis* | *S. cerevisiae* | *A. indonesiensis*  or total bacteria (BTK and GTK) |
| --- | --- | --- | --- | --- |
| BBd7 | 6.5.10^5^ bc | nd | nd | nd |
| HVd7 | nd | 1.4.10^6^ bcd | nd | nd |
| AId7 | nd | nd | nd | 4.2.10^6^ a |
| BBAId7 | 7.9.10^4^ c | nd | nd | 6.8.10^5^ b |
| HVAId7 | nd | 7.1.10^5^ cd | nd | 6.0.10^6^ a |
| BBHVd7 | 1.0.10^6^ bc | 2.0.10^6^ bc | nd | nd |
| Td7 | 1.5.10^6^ bc | 2.4.10^6^ ab | nd | 7.2.10^5^ b |
| BTKd7 | 2.5.10^6^ b | 1.7.10^5^ d | nd | 2.2.10^5^ b |
| GTKd7 | 1.2.10^6^ bc | 3.8.10^5^ d | nd | 2.0.10^4^ b |
| BBd12 | 1.3.10^6^ bc | nd | nd | nd |
| HVd12 | nd | 3.6.10^5^ bcd | nd | nd |
| AId12 | nd | nd | nd | 1.7.10^6^ b |
| BBAId12 | 5.8.10^5^ bc | nd | nd | 4.7.10^5^ b |
| HVAId12 | nd | 1.4.10^6^ bcd | nd | 9.7.10^5^ a |
| BBHVd12 | 8.2.10^5^ bc | 3.3.10^6^ a | nd | nd |
| Td12 | 1.9.10^6^ bc | 3.4.10^6^ a | nd | 1.8.10^6^ b |
| BTKd12 | 9.2.10^6^ a | 1.8.10^5^ d | 9.0.10^3^ a | 2.4.10^5^ b |
| GTKd12 | 8.3.10^6^ a | 7.6.10^5^ cd | 1.0.10^4^ a | 7.7.10^5^ b |
| nd = not detected, inferior to 1.10^3^ CFU mL^-1^. Common letters signify no significant differences between values of the same column according to ANOVA (p < 0.05). BB= *B. bruxellensis*, HV = *H. valbyensis*, SC = *S. cerevisiae*, AI = *A. indonesiensis*, BTK = black tea kombucha, GTK = green tea kombucha. | | | | |

**Supplementary Material S4: Non-volatile chemical parameters in the different cultures at 7 (d7) and 12 days (d12) after inoculation (average values, n = 3).**

| Samples | Sucrose (g L^-1^) | Glucose (g L^-1^) | Fructose (g L^-1^) | pH | Total acidity (meq L^-1^) |
| --- | --- | --- | --- | --- | --- |
| SBT/SGT | 58.3 ± 0.9 | 0.3 ± 0.4 | 0.4 ± 0.4 | 6.90 ± 0.10 | <1 |
| BBd7 | 54.4 a | 0.5 b | 0.4 a | 4.75 a | 4.7 f |
| HVd7 | 55.5 a | 0.9 b | 0.3 a | 4.74 a | 4.7f |
| AId7 | 54.4 a | 2.9 ab | 1.4 a | 4.36 cde | 5.0 f |
| BBAId7 | 55.3 a | 0.7 b | 0.6 a | 4.43 bcd | 9.7 ef |
| HVAId7 | 49.4 ab | 1.0 b | 0.5 a | 4.51 abc | 7.0 f |
| BBHVd7 | 50.3 ab | 1.3b | 0.7 a | 4.68 ab | 6.3 f |
| Td7 | 56.1 a | 0.2 b | 0.2 a | 4.41 bcd | 12.0 de |
| BTKd7 | 50.7 ab | 0.7 b | 0.4 a | 4.09 cdef | 20.7 c |
| GTKd7 | 50.0 ab | 0.2 b | 0.0 a | 4.21 cdef | 19.0 c |
| BBd12 | 45.1 ab | 2.5 ab | 1.1 a | 4.17 defg | 14.0 de |
| HVd12 | 50.7 ab | 2.8 b | 1.2 a | 4.49abc | 8.7 ef |
| AId12 | 43.6 ab | 4.9 a | 2.3 a | 3.87 gh | 13.7 c |
| BBAId12 | 39.1 b | 1.1 b | 0.0 a | 4.04 fg | 20.0 cd |
| HVAId12 | 46.3 ab | 2.0 b | 0.0 a | 3.95 fg | 15.7 cd |
| BBHVd12 | 52.2 ab | 0.1 b | 0.0 a | 4.24 cde | 14.0 de |
| Td12 | 52.0 ab | 0.5 b | 0.4 a | 4.15 defg | 20.0 cd |
| BTKd12 | 42.6 ab | 1.6 b | 0.3 a | 3.69 h | 40.7 a |
| GTKd12 | 47.6 ab | 0.7 b | 0.0 a | 4.00 gf | 28.3 b |
| Common letters signify no significant differences between values of the same column according to ANOVA (p < 0.05). SBT = sugared black tea, SGT = sugared green tea , BB= *B. bruxellensis*, HV = *H. valbyensis*, SC = *S. cerervisiae*, AI = *A. indonesiensis*, BTK = black tea kombucha, GTK = green tea kombucha. | | | | | |

**Supplementary Material S9: Similarity index of the two dendrograms obtained from the sensory scores and the volatile metabolites concentrations for the 32 detected metabolites among the 14 samples as function of k clusters selected.**


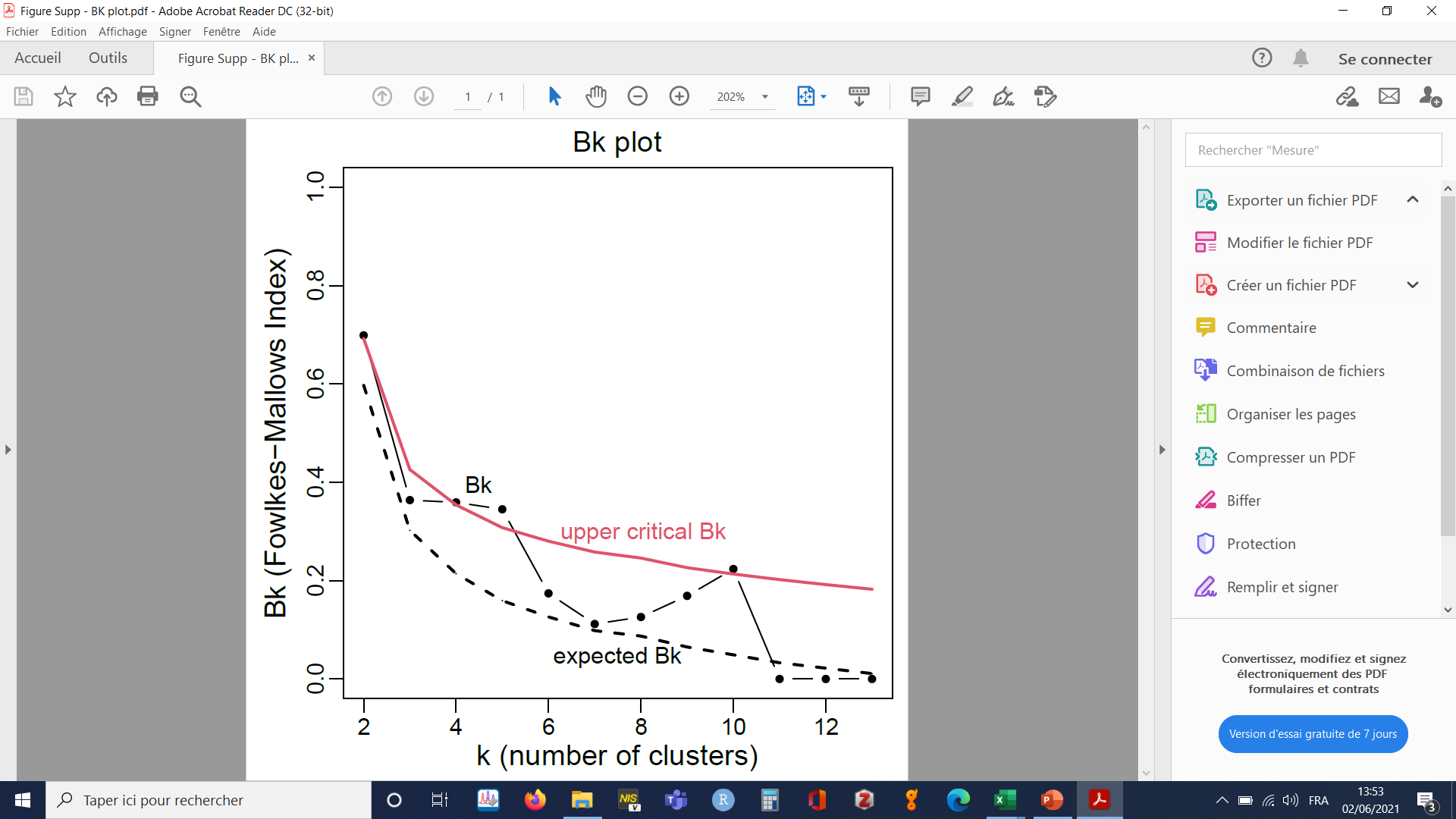

Supplement: Supplementary file 2 [file Data_Sheet_2.docx]
